# Forensic evidence preservation following an incident of rape: The role of the victim

**DOI:** 10.4102/safp.v66i1.5776

**Published:** 2024-01-25

**Authors:** Anthonio O. Adefuye, Chika K. Egenasi, Matthew O.A. Benedict

**Affiliations:** 1 Department of Basic Medical Sciences, College of Dental Medicine, Kansas City University, Joplin, United States of America; 2 Department of Health Sciences Education, Faculty of Health Sciences, University of the Free State, Bloemfontein, South Africa; 3 Department of Family Medicine, Faculty of Health Sciences, University of the Free State, Bloemfontein, South Africa

**Keywords:** rape, rape victim, forensic evidence, forensic examination, evidence preservation, victim’s behaviour, public awareness, South Africa

## Abstract

**Background:**

Rape has a high prevalence in South Africa. The collection of credible and valid forensic evidence is a key legal factor that impacts case trial outcomes. Victim behaviour around the time of the rape can impact the collection and the integrity of forensic evidence, and can have a direct effect on case progression and conviction. Despite the importance of victim behaviour, few studies have been done on the role of victims in preserving forensic evidence. This article discusses how common personal hygiene practices undertaken by rape victims after being raped can impact the quality and validity of forensic evidence. This investigation was done with the aim of elucidating the role of victims in preserving forensic evidence post rape.

**Methods:**

This was a descriptive, retrospective clinical audit of all rape victims managed at Robert Mangaliso Sobukwe Hospital forensic unit in South Africa from 01 January 2020 to 31 March 2022.

**Results:**

A total of 192 rape cases over the study period were included in this review. The median age of rape victims was 20 years (minimum 2 years; maximum 76 years). The majority (*n* = 178; 92.7%) of the victims were female. About 44.8% (*n* = 86) of the victims reported that they had urinated post-rape and prior to forensic examination, 20.8% (*n* = 40) had changed their clothing, 8.3% (*n* = 16) had showered, 6.8% (*n* = 13) had bathed, 4.2% (*n* = 8) had douched, and only 1.0% (*n* = 2) had defecated. Only 44 (22.9%) of the victims reported to have ingested alcohol or spiked drinks before the rape.

**Conclusion:**

These findings suggest that some rape victims engaged in personal hygiene practices that could militate against forensic evidence preservation. This finding, therefore, indicates the need for public awareness about ways to preserve evidence to the greatest extent possible after an incident of rape.

**Contribution:**

We provide simple guidelines for victims on the preservation of forensic evidence following rape and before detailed forensic medical examination and evidence collection.

## Introduction

South Africa has one of the highest incidences of rape globally and has been called ‘the rape capital of the world’.^1^ The reported rape rate in South Africa was 72.1/100 000 in 2019–2020.^2^ According to the South African Police Service (SAPS) crime statistics report, 10 512 rape cases were reported in the first quarter of 2023.^3^ Most cases of rape in South Africa go unreported, because of a lack of faith in the criminal justice system and medical services and fear of secondary trauma.^1^ Victims of rape are usually women and girl children – very few rape cases involving men are reported, possibly because of fear of stigma and prevailing gender stereotypes.^4,5^ Most rapes are perpetrated by men, and population-based surveys in parts of South Africa report that up to a third of men disclosed having perpetrated rape and close to a fifth disclosed having done so repeatedly.^6,7^

Harmful cultural practices and beliefs that undermine the rights of women and girl children, the high rate of unemployment, social inequality, substance abuse, and patriarchal beliefs have been established as enablers of rape in the country.^8,9^ While a range of measures intended to combat the high incidence of rape and improve the management of rape survivors has, over the years, been introduced by the South African government, successful conviction rates for rape crimes remain low.^10^ A combination of legal and extra-legal factors have been implicated in the attrition of rape cases within the South African criminal justice system.^11^ Machisa et al.^11^ report that the availability of credible forensic evidence is a key legal factor for case trial outcomes. Using a Sexual Assault Evidence Collection Kit (SAECK) within 96 h of a rape incident, proper collection and preservation of DNA samples and successful matching of the perpetrator’s DNA can increase the credibility of forensic evidence and double the likelihood of guilty convictions.^11^ Reported extra-legal factors include victim characteristics (e.g., race), relationship with the perpetrator (e.g., being an intimate partner, a stranger or related to the perpetrators), and victim behaviour (e.g., alcohol or other intoxication).^11^

Collecting forensic evidence and maintaining a chain of evidence after an incident of rape is a complex and delicate process.^12^ The success of this process is dependent on the first responder (e.g. the first police officer or investigating officer arriving at the scene), the medical examiner (accredited healthcare practitioner), and the victim’s behaviour around the time of the rape.^11^ If forensic evidence is collected, handled and processed ineptly by the healthcare professional or investigating officer, the evidence may be inadmissible in a court of law.^10^ Similarly, the quality and validity of forensic evidence are critically affected both physically and chemically by actions taken by the victim immediately post rape and by the passage of time.^13,14^ Personal hygiene practices, such as bathing, changing clothing, and douching can have a negative impact on the credibility of forensic evidence.^15,16^ While discussions in numerous studies are centred on educating healthcare professionals and personnel of the SAPS on the preservation of forensic evidence, no mention is made of the importance of the victims, or their role in preserving possible forensic evidence.^17,18,19^ In this short scientific report, we discuss how common personal hygiene practices by rape victims, post rape, can impact the quality and validity of forensic evidence. This is done with the aim of elucidating the important role of victims in the preservation of forensic evidence. We provide simple guidelines on the preservation of forensic evidence after an incident of rape and before a detailed forensic medical examination is undertaken and evidence is collected by a dedicated healthcare practitioner.

## Methodology

This was a descriptive, retrospective clinical audit of all rape victims managed at Robert Mangaliso Sobukwe Hospital (RMSH) forensic unit from 01 January 2020 to 31 March 2022.

### Study setting

Kimberley hospital, now RMSH, is a regional and tertiary hospital in the Northern Cape province; it is the only referral centre in the Northern Cape and receives referred patients from all over the province. Similarly, the RMSH forensic department is a referral centre for children younger than ≤ 13 years and adults who have been victims of rape in the province.

### Data collection

Information on all rape victims managed at the RMSH forensic department from 01 January 2020 to 31 March 2022 was retrieved from the patient register in the forensic department. The pertinent case records were subsequently recovered from the records department. A datasheet was designed by one of the researchers, based on trends observed in similar study, and was used to collect data from the patients’ clinical notes, and the J88 form (medico-legal report).^20^ The datasheet was pretested on the first 10 files (in succession). This was done to ensure that the variables on the datasheet were well and correctly structured. It also ensured that data were captured accurately and quality was maintained. No significant changes resulted from the pretesting, and the data obtained from the 10 patient files were included in overall data of the study. Data obtained from the patient files include age, gender, use of recreational substances, and reported personal hygiene practices.

#### Inclusion criteria

The study inclusion criteria were:

- positive history of rape regardless of age
- patient brought in by a law enforcement officer who had completed the SAPS 308 form (authorisation form)^20^
- patient examined and managed at the RMSH forensic unit, with duly completed J88 form.^20^

#### Exclusion criteria

Patients for whom no evidence had been collected, incomplete medical records, and no J88 form completed.

### Data analysis

Data were entered into an Excel spreadsheet (Microsoft Office Professional Plus 2016) and analysed using the Statistical Package for Social Sciences (IBM SPSS Statistics 25).

### Ethical considerations

The Northern Cape Department of Health approved the protocol for the study and issued approval (No. NC_2022RMSH04_001). No patient names or personal identifiers appeared in the data collection forms. All patient records were stored in a secure location and were only available to one of the researchers (C.K.E.), who collated the data. All patient information and records were managed in a strictly professional and confidential manner.

## Results

A total of 192 rape cases were included in this review over the study period. The age range of victims was from 2 years to 76 years, with a median age of 20 years. The majority (178; 92.7%) of the victims were female.

### Use of recreational substances

Only 22.9% (*n* = 44) of the victims reported to have ingested alcohol or spiked drinks before the rape. No other recreational substances were reported to have been used by the victims.

### Personal hygiene practices reported by the victims

About 44.8% (*n* = 86) of the victims reported that they had urinated post rape and prior to forensic examination, 20.8% (*n* = 40) had changed their clothing, 8.3% (*n* = 16) had showered, 6.8% (*n* = 13) had bathed, 4.2% (*n* = 8) had douched, and only 1.0% (*n* = 2) had defecated.

## Discussion

Drug-facilitated rape is a prevalent phenomenon that is often underreported. Smith et al.^21^ reported in 2018 that 11% of adult women in the United States (US) had been raped at some time in their lives while they were incapacitated by alcohol or other drugs. Similarly, 38% of adult female rape victims in the United Kingdom (UK) reported having been raped while they were under the influence of alcohol.^22^ There is a paucity of epidemiological and toxicological data associated with drug-facilitated rape in South Africa.^23^ Young women are often the victims of drug-facilitated rape; ethanol is reported to be the most commonly involved drug.^23^ Other common drugs used include methamphetamine, methaqualone and diphenhydramine, either alone or in combinations.^23^ Opportunistic drug-facilitated rape, an act in which a victim is rendered incapable of giving consent after voluntary consumption of alcohol and/or pharmacological agent (medicinal and/or recreational), has been reported as involving most cases of rape.^24^ Proactive drug-facilitated rape, wherein the victim’s incapacity results from involuntary consumption, has also been reported.^24^ Our findings were that 22.9% of victims reported using alcohol or spiked drinks prior to rape, which confirms findings of a study done in Cape Town by Oshodi et al.,^25^ wherein rape survivors reported using alcohol prior to being raped. Analysing urine samples is of the utmost importance in drug-facilitated rape, in which case the first available urine sample should be collected.^26^ An early submission of urine specimen by the victim post rape increases the chance of detecting drugs that are eliminated from the body quickly. Victims should not urinate until after evidence has been collected, and the number of times a victim urinated before the sample was collected should be documented.^27^ The majority of victims in our study (44.8%) reported having urinated post rape and prior to forensic examination, thus, making it improbable that credible evidence can be collected if they had been a victim of drug-facilitated rape. Urine samples allow for a longer detection time than blood samples.^27^ A urine specimen of at least 30 millilitres, preferably 100 millilitres, should be collected in a clean plastic or glass container.^28^ The urine sample does not have to be a clean catch (i.e. bacteria in the urine will not compromise test results). If a victim cannot wait to reach the examination facility before urinating, the victim should be asked to provide a sample and bring it to the facility, and the chain of evidence documented.^27^ The lack of experience of role players dealing with the investigation and management of drug-facilitated rape has been reported as one of the numerous factors that have an impact on the successful investigation of these cases.^23^ Training and empowerment of role players and subsequent public health education and policy development are, therefore, essential.

It has been reported that clothing frequently contains the most important evidence in cases of rape.^29^ Clothing provides a surface upon which traces of foreign material, such as the perpetrator’s semen, saliva, blood, hairs, fibres, and debris from the crime scene may be found. Damaged or torn clothing may be significant and provide evidence of force.^17^ Our findings show that some victims (20.8%) changed their clothing before being examined. This is consistent with the findings by Hassan et al.,^15^ who reported that rape victims in their study changed their clothes before medical examination. Changing of clothing prior to examination may adversely affect or lead to the loss of credible forensic evidence. The case in its entirety may be jeopardised if clothing was discarded and not brought forward for forensic examination. Victims should be advised to preserve as much physical evidence as possible, and to not discard the clothing worn during the incident. Garments should be kept separate from one another, to allow forensic scientists to reach pertinent conclusions regarding the reconstruction of criminal actions.^29^

During an act of rape, the perpetrator may leave DNA evidence on the surface of the victim’s body. Body fluids such as semen, blood, saliva or sweat are DNA-rich evidence that can be deposited on a victim’s body. Sanitary practices such as bathing or showering may negatively affect DNA recovery.^30^ Our findings reveal that some victims (15.1%) in our study, reported bathing or showering prior to examination. This confirms findings by Patra et al.,^16^ who reported that victims of rape in their study reported taking a bath or washing their bodies before the medical examination. This suggests that some of the victims are ignorant about the implications of such practices for forensic evidence. Victims should be informed that they should not bath or shower before presenting for examination. Early presentation should be advocated for, as DNA evidence on the surface of the victim’s body may become unrecoverable after a short time.^30^

Forensic examination and collection of DNA specimens from the female genitalia are done in cases that involve vaginal penetration. A high level of prostatic-specific acid phosphatase, an enzyme found in large quantities in seminal fluid, in the vaginal specimen indicates recent sexual contact.^31^ Douching has a chemical effect on the quantity and quality of semen remaining in the vagina.^31^ This suggests that, if victims report vaginal penetration, the integrity of DNA specimens obtained from victims who reported to have douched (4.2%) may be compromised. Failure to explain the circumstances under which semen could have been destroyed could jeopardise criminal prosecution if apparent contradictions cannot be accounted for in court.^29^ Furthermore, defecation could negatively impact the quality and quantity of semen or DNA-rich samples remaining in the anal orifice in suspected cases of anal penetration.

## Conclusion

**FIGURE 1:**
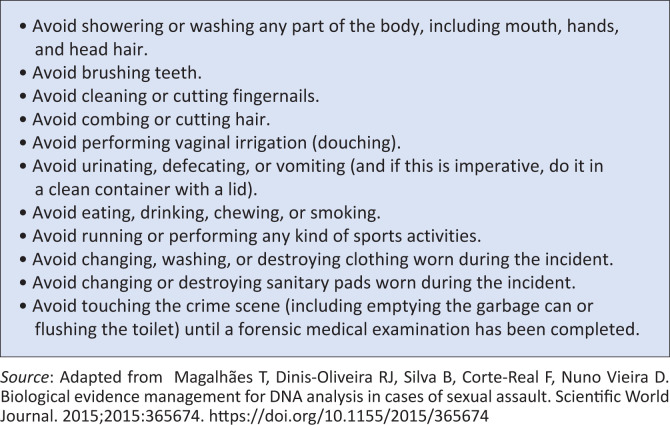
Guidelines for the preservation of forensic evidence after an incident of rape.

Using a descriptive, retrospective clinical case audit, we obtained data on the personal hygiene practices reported by victims post rape, and discussed how these practices can impact the quality and validity of forensic evidence. These findings suggest that some rape victims engaged in hygiene practices that could militate against forensic evidence preservation. This finding, therefore, indicates the need for public awareness on how to preserve evidence to the greatest extent possible after an incident of rape. The general public are enjoined to follow the simple guidelines listed in Figure 1 to preserve the integrity of possible forensic evidence. We believe that these guidelines can be used to enlighten the South African public on how to preserve forensic evidence after an incident of rape. Applying these guidelines could invariably reduce the attrition of rape cases and increase the likelihood of convictions by the South African criminal justice system.

Law enforcement agencies and health professions bodies should establish detailed guidelines to enlighten the South African public on how to ensure that the integrity of possible forensic evidence is maintained after an incident of rape. Established guidelines can be published as pamphlets in all 12 official South African languages and disseminated to schools and public arenas.
